# Microbial tryptophan catabolites in health and disease

**DOI:** 10.1038/s41467-018-05470-4

**Published:** 2018-08-17

**Authors:** Henrik M. Roager, Tine R. Licht

**Affiliations:** 1 Department of Nutrition, Exercise and Sports, Faculty of Science, University of Copenhagen, DK-1958 Frederiksberg, Denmark; 2 National Food Institute, Technical University of Denmark, DK-2800 Kgs Lyngby, Denmark

## Abstract

Accumulating evidence implicates metabolites produced by gut microbes as crucial mediators of diet-induced host-microbial cross-talk. Here, we review emerging data suggesting that microbial tryptophan catabolites resulting from proteolysis are influencing host health. These metabolites are suggested to activate the immune system through binding to the aryl hydrocarbon receptor (AHR), enhance the intestinal epithelial barrier, stimulate gastrointestinal motility, as well as secretion of gut hormones, exert anti-inflammatory, anti-oxidative or toxic effects in systemic circulation, and putatively modulate gut microbial composition. Tryptophan catabolites thus affect various physiological processes and may contribute to intestinal and systemic homeostasis in health and disease.

## Abstract (web summary)

Gut microbial metabolites are known to impact many physiological processes of the host and play a critical role in immune-homeostasis. Here the authors review our current understanding and appreciation of the importance of microbially derived tryptophan catabolites during both health and disease.

## Introduction

**Fig. 1:**
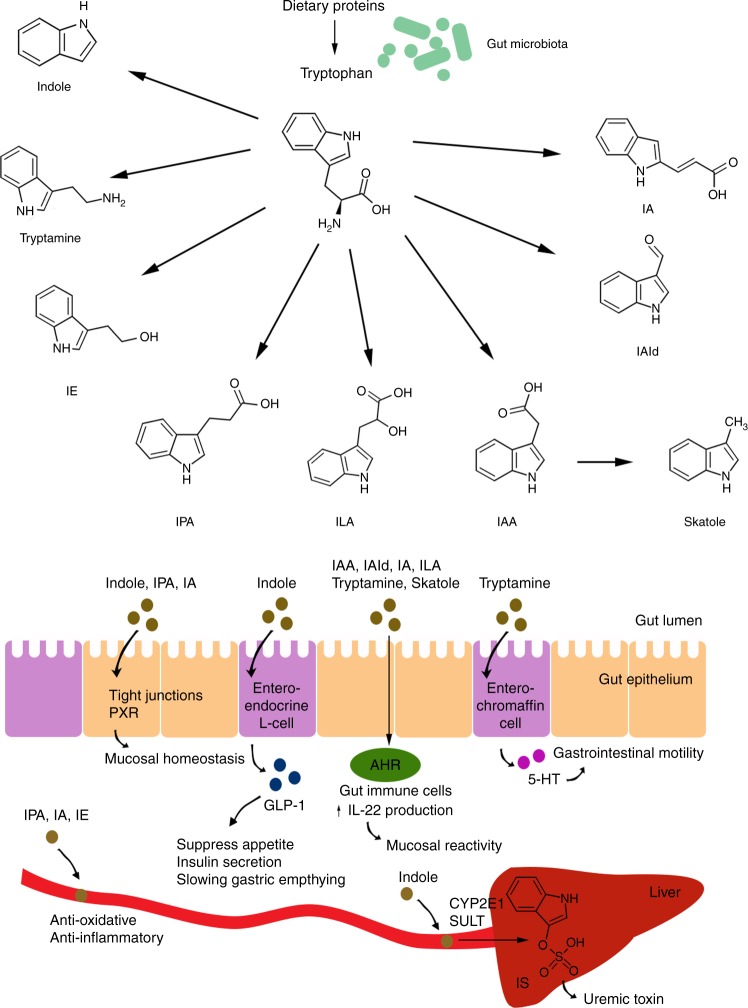
Mechanisms of action of microbial tryptophan catabolites on host physiology. Degradation of dietary proteins leads to the release of tryptophan, which is converted into various catabolites by the gut microbiota. The tryptophan catabolites include indole, tryptamine, indoleethanol (IE), indolepropionic acid (IPA), indolelactic acid (ILA), indoleacetic acid (IAA), skatole, indolealdehyde (IAld) and indoleacrylic acid (IA) and may affect host physiology in numerous ways. Indole, IPA and IA affect mucosal homeostasis by decreased intestinal permeability possibly mediated by the pregnane X receptor (PXR). Indole induces the release of glucagon-like peptide 1 (GLP-1) in enteroendocrine L-cells. GLP-1 is known to suppress appetite, insulin secretion and to slow gastric emptying. Several tryptophan catabolites act on the aryl hydrocarbon receptor (AHR) found in intestinal immune cells and thereby alter innate and adaptive immune responses in a ligand-specific fashion (e.g. IAld induces increased production of interleukin-22 (IL-22) via AHR activation). Tryptamine induces the release of 5-hydroxytryptamine (5-HT, serotonin) by enterochromaffin cells. 5-HT stimulates gastrointestinal motility by acting on enteric nervous system neurons. Tryptophan catabolites are absorbed through the intestinal epithelium and enter the bloodstream where some (e.g. IPA, IE, IA) have anti-oxidative and anti-inflammatory effects, whereas indoxyl-sulfate (IS), which is produced in the liver from indole by the actions of CYP2E1 and sulfotransferases (SULT), has cytotoxic effects in high concentrations

The diverse and dynamic microbial community of the human gastrointestinal tract plays a vital role in health and nutrition of the host^1^. A mutualistic relationship between host and gut microbiota relies on complex molecular cross-talk, which is fundamental for intestinal homeostasis. Although recent advances in characterizing the composition and function of the gut microbiota have yielded numerous new findings about the role of the gut microbiota in human health, only few microbiota-generated metabolites affecting host physiology have been identified, but these include short-chain fatty acids (SCFA) originating from bacterial degradation of dietary fiber^2^, secondary bile acids originating from bacterial conversion of bile acids in the colon^3^, and trimethylamine-N-oxide (TMAO), which is a product of microbial-host co-metabolism of nutrients such as phosphatidylcholine, choline, and *L*-carnitine, present in high-fat foods^4,5^. The role of bacterial metabolites originating from proteolysis on host physiology has however received only scarce attention. Although bacterial protein degradation products in general have been considered to be deleterious for the host^6^, recent data suggests that tryptophan catabolites generated by the gut microbiota are important contributors to intestinal homeostasis. Here, we review recent discoveries related to microbial tryptophan catabolites and discuss future efforts to explore their potential role in mediating microbe-host interactions (Fig. 1). Since available studies linking tryptophan catabolites with health are typically associative or originate from mouse models, more research is needed to provide tangible connections between tryptophan catabolites and human health.

## Bacterial tryptophan catabolism in the gut

**Fig. 2:**
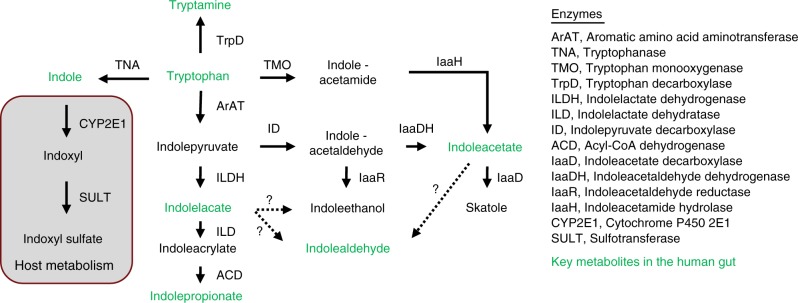
Microbial generation of tryptophan catabolites in the human gut. Overview of the different ways microbes degrade tryptophan in the human gut. The metabolites that are most often found in the human gut are colored in green. Dashed lines represent pathways where no enzymes have been identified

Tryptophan is an essential amino acid for humans, and is supplied by dietary protein (Box 1). Although the majority of ingested protein is digested and absorbed in the small intestine^7^, dependent on intake, significant amounts of proteins and amino acids (6–18 g/day) may reach the colon^8^, where a range of commensal bacteria degrade them. Bacterial protein catabolism increases with increased protein intake, carbohydrate depletion in the colon, increased colonic pH and prolonged colonic transit time^9–12^. The gradual depletion of carbohydrate substrates occurring from the proximal to the distal colon causes a shift in bacterial catabolism from saccharolytic to proteolytic fermentation^10^. In agreement herewith, concentrations of phenolic compounds from degradation of aromatic amino acids in the human gut content are more than fourfold higher in the distal colon than in the proximal colon^12^. Bacterial proteolytic specialists are reported to have lower growth potential than generalists and saccharolytic/lipolytic specialists, suggesting that proteolytic specialists thrive when the ecological pressure for fast growth is decreased^13^. However, the degradation of tryptophan appears not to be limited to proteolytic specialists or to the distal colon as for example the Lactobacilli catabolize tryptophan in the stomach and ileum of mice^14^. Several bacterial species have been reported to be able to convert tryptophan into indole and indole derivatives (Table 1). Already in 1897, tryptophan was found to be converted into indole by *Bacillus coli* (now *Escherichia coli*) and *Asiatic cholera* (now *Vibrio cholerae*)^15^. Later, the production of indole has been used as a diagnostic marker to distinguish *E. coli* from other enteric bacteria. Because indole has been known for more than 100 years, numerous indole-producing species have been identified as described in a previous review^16^; therefore these are not discussed in details here. Briefly, indole formation occurs via the action of the enzyme tryptophanase (TnaA), which is expressed in many Gram-negative, as well as Gram-positive bacterial species including *Escherichia coli, Clostridium* spp. and *Bacteroides* spp.^12,16–18^. More recently, a number of studies have shown that gut microbial species produce a variety of tryptophan catabolites via various other metabolic pathways (Fig. 2). For example, *Clostridium sporogenes* converts tryptophan into tryptamine, indolelactic acid (ILA) and indolepropionic acid (IPA)^19–21^. Likewise, *Peptostreptococcus* spp. including *P*. *russellii, P. anaerobius* and *P. stomatis* are known to convert tryptophan to indoleacrylic acid (IA) and IPA, possibly due to the presence of the phenyllactate dehydratase gene cluster (*fldAIBC*) on the chromosome of these species^22^. Indeed, a homologue of this cluster is found to be responsible for the conversion of tryptophan into ILA and IPA in *C. sporogenes*^21^. Furthermore, homologue gene-clusters were found in *Clostridium cadaveris*, *Clostridium botulinum* and *Peptostreptococcus anaerobius*^21^ in agreement with their capability to produce IPA^17,21,22^. Another group of bacteria capable of converting tryptophan is Lactobacilli. *Lactobacillus* spp. convert tryptophan to indolealdehyde (IAld) and ILA via the aromatic amino acid aminotransferase (ArAT) and an indolelactic acid dehydrogenase (ILDH)^14,23,24^. *Ruminococcus gnavus* converts tryptophan into tryptamine by the action of a tryptophan decarboxylase enzyme^20^. Several *Bacteroides* species, as well as *Clostridium bartlettii* have been reported to produce ILA and indoleacetic acid (IAA)^25^, whereas *Bifidobacterium* spp. have been reported to produce ILA^25,26^. Finally, the common intestinal metabolite 3-methylindole (skatole), which have been extensively studied as the cause of off-flavor in pork, is generated by decarboxylation of IAA by *Bacteroides* spp. and *Clostridium* spp.^12,17,25,27^. Although several species are able to metabolize tryptophan in vitro (Table 1), studies linking the abundances of bacterial species with concentrations of tryptophan catabolites in humans are still needed to identify the main tryptophan catabolite producers in the human gut. Indole and IAA are detected in human fecal samples of healthy adults at mean concentrations of 2.6 mM^28^ and 5 µM^29^, respectively. However, the concentrations of other tryptophan catabolites (IPA, ILA, IAld, tryptamine and IA) in the human gut have to our knowledge not been assessed. In serum, mean concentrations in healthy adults have previously reported for IAA (1.3 µM), IPA (1.0 µM), and ILA (0.15 µM)^30^, while a recent study reported a mean serum IPA concentration of 50 nM^31^. Additionally, the mean concentrations of microbial tryptophan catabolites in the urine of pregnant women were recently reported for IAA (61 µM), methyl-IAA (8 µM), tryptamine (9 µM), and methyl-IPA (0.5 µM)^32^. Despite differences in the reported concentrations, these studies suggest that in adults, indole is the most abundant microbial tryptophan catabolite, followed by IAA and IPA. The availability of methods for determination of concentrations of microbial tryptophan catabolites in biological specimens is however currently limited^28,32^ and better quantitative analytical methods targeting a larger variety of microbial tryptophan metabolites are needed. Such methods will enable the comparison of metabolite concentrations across biological compartments (i.e. feces, blood and urine) and between different human populations.

**Table 1:** Gut bacterial species reported to produce tryptophan catabolites

Metabolite	Producers		References
Indole	*Bacteroides thetaiotaomicron* *Bacteroides ovatus* *Clostridium limosum* *Clostridium bifermentans* *Clostridium malenomenatum* *Clostridium lentoputrescens* *Clostridium tetani* *Clostridium tetanomorphum*	*Clostridium ghoni* *Clostridium sordellii* *Desulfovibrio vulgaris* *Enterococcus faecalis* *Escherichia coli* *Fusobacterium nucleatum* *Haemophilus influenza* *Peptostreptococcus asscharolyticus* … for more see^16^	12,16–18
3-methylindole (Skatole)	*Bacteroides thetaiotaomicron* *Butyrivibrio fibrisolvens* *Clostridium bartlettii* *Clostridium scatologenes* *Clostridium drakei*	*Eubacterium cylindroides* *Eubacterium rectale* *Lactobacillus* spp. *Megamonas hypermegale* *Parabacteroides distasonis*	25,27,118
Indoleacetic acid (IAA)	*Bacteroides thetaiotaomicron* *Bacteroides eggerthii* *Bacteroides ovatus* *Bacteroides fragilis* *Bifidobacterium adolescentis* *Bifidobacterium longum* subsp. *longum* *Bifidobacterium pseudolongum* *Clostridium bartlettii* *Clostridium difficile* *Clostridium lituseburense* *Clostridium paraputrificum*	*Clostridium perfringens* *Clostridium putrefaciens* *Clostridium saccharolyticum* *Clostridium sticklandii* *Clostridium subterminale* *Escherichia coli* *Eubacterium hallii* *Eubacterium cylindroides* *Parabacteroides distasonis* *Peptostreptococcus asscharolyticus*	12,17,25
Indoleacrylic acid (IA)	*Clostridium sporogenes* *Peptostreptococcus russellii* *Peptostreptococcus anaerobius* *Peptostreptococcus stomatis*		21,22
Indolealdehyde (IAld)	*Lactobacillus acidophilus* *Lactobacillus murinus* *Lactobacillus reuteri*		14,23,24
Indoleethanol (IE)	*-*		-
Indolelactic acid (ILA)	*Anaerostipes hadrus* *Anaerostipes caccae* *Bacteroides thetaiotaomicron* *Bacteroides eggerthii* *Bacteroides ovatus* *Bacteroides fragilis* *Bifidobacterium adolescentis* *Bifidobacterium bifidum* *Bifidobacterium longum* subsp. *infantis* *Bifidobacterium longum* subsp. *longum* *Bifidobacterium pseudolongum* *Clostridium bartlettii* *Clostridium perfringens*	*Clostridium sporogenes* *Clostridium saccharolyticum* *Escherichia coli* *Eubacterium rectale* *Eubacterium cylindroides* *Faecalibacterium prausnitzii* *Lactobacillus murinus* *Lactobacillus paracasei* *Lactobacillus reuteri* *Megamonas hypermegale* *Parabacteroides distasonis* *Peptostreptococcus asscharolyticus*	12,21,23–26,42
Indolepropionic acid (IPA)	*Clostridium botulinum* *Clostridium caloritolerans* *Clostridium paraputrificum* *Clostridium sporogenes* *Clostridium cadvareris*	*Peptostreptococcus asscharolyticus* *Peptostreptococcus russellii* *Peptostreptococcus anaerobius* *Peptostreptococcus stomatis*	17,19–22
Tryptamine	*Clostridium sporogenes* *Ruminococcus gnavus*		20

### Box 1 Some facts about tryptophan

- Tryptophan is one of the nine so-called essential amino acids, which the human organism cannot synthesize, and which therefore must be supplied in the diet.
- Tryptophan is the only amino acid that contains the structure of an indole, i.e. a bicyclic compound, consisting of a six-membered benzene ring fused to a five-membered, N- containing, pyrrole ring.
- Tryptophan is a part of dietary proteins, and is thus high in protein-rich foods such as meat, fish, eggs, cheese, beans, and nuts.
- Tryptophan is taken up in the small intestine, but the fraction that reaches the colon can be catabolized by the gut bacteria resulting in a variety of indole-derivatives (Fig. 1).
- While bacterial products of protein degradation are generally associated with detrimental effects, new research suggests that microbial tryptophan catabolites may also have a positive impact on host physiology.

## Mechanisms of action of microbial tryptophan catabolites

A growing body of literature suggests that tryptophan catabolites generated by the gut microbiota are important signaling molecules in microbial communities, as well as in host-microbial cross-talk, and may contribute to intestinal and systemic homeostasis (Fig. 1).

## Microbial benefits of tryptophan metabolism

While the interaction of intestinal bacterial tryptophan metabolites with the host has received an increasing amount of attention in recent years as reviewed in the following sections, knowledge about the benefit of tryptophan metabolism to the intestinal microbes themselves is limited. Tryptophan has a multitude of metabolic functions, for example, it is incorporated into polypeptide chains of bacterial enzymes and serves as a precursor of the cofactor NAD^33^. However, most important in the rich intestinal environment is probably the bacterial need for maintenance of redox homeostasis. In the gut, the bacteria generate ATP from fermentation of carbohydrates, which typically involves oxidative steps. Compensating reductions must thus be carried out. As recently reviewed for food fermentation involving lactic acid bacteria^34^, microbes apply a multitude of different reactions in order to find ultimate electron acceptors allowing them to balance their fermentative metabolism in the absence of oxygen. Similarly in the gut, indole pyruvate generated by transamination of tryptophan may thus be applied as electron acceptor and reduced to ILA by action of the ILA dehydrogenase (Fig. 2). Such fine-tuning reactions might thus equilibrate the redox potential and give microbes a competitive advantage in the gut. Additionally, bacterial cross-feeding on degradation products of aromatic amino acids is likely to occur in the human colon^35^, and may provide specific bacteria with a growth advantage in the highly competitive gut ecosystem.

## Tryptophan catabolites as signals and antimicrobial agents

Indole is well-described as an intercellular signal molecule that appears to be important in microbial communities by affecting spore formation, plasmid stability, drug resistance, biofilm formation, and virulence^16^. Similarly, indoleethanol (IE, tryptophol) has been identified as a quorum sensing molecule in fungi^36^. This metabolite also exerts antimicrobial activity towards *Staphylococcus aureus*, *Salmonella enterica* and *Lactobacillus plantarum*^37,38^. Moreover, IE inhibits bacteriophage replication in a thermophilic bacterium, *Geobacillus* sp. E263^39^, virus replication in shrimps^40^, and proliferation of parasitic protozoa^41^. Also ILA is reported to have antifungal activity against *Penicillium* strains^42^ and anti-bacterial activity against *E. coli* and *B. cereus*^43^. Furthermore, there is evidence suggesting that indoles affect the survival of the nematode *Caenorhabditis elegans*^44,45^. Collectively, these diverse examples suggest that indoles play a role as modulators of microbial gut communities across kingdoms including bacteria, fungi and viruses. Yet, whether the tryptophan catabolites modulate also the microbial community of the mammalian gut remains unexplored.

## Tryptophan catabolites as aryl hydrocarbon receptor ligands

In recent years it has become evident that bacterial tryptophan catabolites including tryptamine, skatole, IAA, IA, IAld, and ILA are acting as ligands of the aryl hydrocarbon receptor (AHR)^14,23,46,47^. AHR is a transcription factor widely expressed by cells in the immune system^48^ and a number of studies have demonstrated that AHR activation alters innate and adaptive immune responses in a ligand-specific fashion^49–51^. Importantly, the affinities of tryptophan catabolites for the AHR differ between mice and humans^47^ and even within laboratory mouse strains, where four alleles encoding different forms of the AHR are known to exist^52^. Whereas the rodent AHR has been found to bind the exogenous ligand 2,3,7,8-tetrachlorodibenzo-p-dioxin (TCDD or dioxin) with approximately 10-fold higher affinity than the human AHR^53^, recent studies suggest that the human AHR has higher affinity than the mouse AHR for a number of tryptophan-derived ligands^47^. Together, these studies reveal that rodent AHR and human AHR exhibit different ligand selectivity, which is very important to consider given that predictions about AHR ligand-receptor interactions in humans are often based on rodent studies. Furthermore, in intestinal cell line models, the tryptophan catabolites have been found to be either agonists or antagonists depending on their molecular structure^46,54^. Recent studies have underlined that indole-induced AHR activation may be one way that bacteria contribute to mucosal homeostasis. *Lactobacillus* spp. were found to regulate interleukin (IL)-22 mucosal homeostasis via IAld-mediated activation of AHR, and thereby to protect mice against mucosal candidiasis^14^. In addition, treatment of mice with three *Lactobacillus* strains capable of metabolizing tryptophan attenuated intestinal inflammation via AHR activation, as the effects were abrogated in the presence of an AHR antagonist^29^. Also *Lactobacillus bulgaricus* OLL1181 was found to activate the AHR pathway and inhibit colitis in a mouse model^55^. Further deciphering the mechanisms, a recent study in mice revealed that *Lactobacillus reuteri* via IAld and ILA activates AHR, which reprogram intraepithelial CD4+ T helper cells into immunoregulatory T cells (CD4+ CD8αα double-positive intraepithelial lymphocytes)^23^. Additionally, ILA was found to inhibit mouse polarization of T helper 17 (T_H_17) cells in vitro^24^. Together these studies suggest that tryptophan catabolites via AHR affect the differentiation of naive CD4+ T helper cells into regulatory T (Treg) cells and T_H_17 cells. The T_H_17/Treg balance plays an important role in autoimmune and inflammatory diseases^56^. Thus, the discovery of tryptophan catabolites as AHR ligands may provide new insight about how microbial metabolites affect the immune system in the gut, as well as in systemic circulation. In addition, indoles provided by commensal bacteria have been found to improve the health of a range of different organisms including *Caenorhabditis elegans, Drosophila melanogaster* and mice in an AHR-dependent way^57^. This raises the intriguing possibility that tryptophan catabolites via AHR may reduce frailty and improve health also in humans.

## Tryptophan catabolites and intestinal barrier functions

Both in vitro and in vivo studies have indicated that indole enhances intestinal epithelial barrier functions by increasing expression of genes involved in maintenance of epithelial cell structure and function^58,59^. Moreover, also IPA was found to regulate intestinal barrier function in vivo in mice by acting as a ligand for the xenobiotic sensor, pregnane X receptor (PXR), particularly in the presence of indole^60^. Activation of PXR has been shown to protect the barrier function in a mouse model of colitis^61^. In addition, IPA was found to reduce intestinal permeability in mice fed a high fat diet^62^. Further emphasizing the link between IPA and intestinal barrier function, a recent study colonized germ-free mice with either a wild-type or *fldC* mutant *C. sporogenes* (IPA production in *C. sporogenes* require an intact *fldC* gene), and showed that the *fldC*-colonized mice exhibited significantly increased permeability to FITC-dextran compared to their wild-type-colonized counterparts, concordant with depleted levels of luminal IPA^21^. Also IA has recently been shown to promote intestinal epithelial barrier function and mitigate inflammatory responses in mice by promoting goblet cell differentiation and mucus production, possibly mediated by AHR activation^22^. Collectively, these studies suggest that tryptophan catabolites signal through PXR and AHR to fortify the intestinal epithelial barrier function.

## Tryptophan catabolites and gut hormone secretion

Indole has been seen to function as a signaling molecule, which is able to modulate the secretion of glucagon-like peptide-1 (GLP-1) from immortalized and primary mouse colonic enteroendocrine L cells^63^. GLP-1 plays a critical role in stimulating insulin secretion from pancreatic beta cells, suppressing appetite and slowing gastric emptying^64^. Thus, intestinal levels of indole may this way affect appetite. Higher serum concentrations of IPA have recently been associated with reduced prevalence of type 2 diabetes and better insulin secretion and sensitivity^65^, which adds to the evidence pointing towards a putative role of indoles in modulating glucose metabolism, possibly via L cell-induced secretion of GLP-1. In line with this, a recent study found that rats fed a diet containing IPA had significantly lower fasting blood glucose level compared to rats fed a control diet^66^. Although the sensing of indole derivatives by L-cells remains elusive, it seems plausible that G protein-coupled receptors (GPCRs), which are responsive to a range of nutrients and other food components^67^, may also be responsive to microbial metabolites including tryptophan catabolites as reviewed elsewhere^68^. Nonetheless, these studies call for more research to be done to elucidate how tryptophan catabolites may modulate the enteroendocrine system and metabolic homeostasis including glucose metabolism.

## Tryptamine and gastrointestinal motility

Tryptamine, a tryptophan catabolite produced by *C. sporogenes and Ruminococcus gnavus*^20^, is a β-arylamine neurotransmitter, which may influence gut health. In the gut, tryptamine is known to induce the release of the neurotransmitter 5-hydroxytryptamine (5-HT, serotonin) by enterochromaffin cells^69^, which are located at the mucosal surfaces^70^. 5-HT stimulates gastrointestinal motility by acting on enteric nervous system neurons^70^. In addition, using an Ussing chamber with a segment of proximal-mid murine colon mucosa, it was found that tryptamine itself induced a significant change in short circuit current, confirming that it can affect ion secretion in intestinal epithelial cells^20^, which plays an important role in gastrointestinal motility. Thus, tryptamine may act as a signaling molecule that affects intestinal transit time, which is strongly associated with the gut microbial composition, diversity and metabolism in humans^10,71^. Whether bacterial production of tryptamine plays a role in the pathogenesis of irritable bowel syndrome, which often manifests as either chronic diarrhea or chronic constipation^72^, currently remains unanswered. One species of particular interest could be *R. gnavus*, which is over-represented in inflammatory bowel disease (IBD)^73,74^, produces tryptamine^20^ and utilizes mucin^75^, suggesting that it thrives in close proximity with the enterochromaffin cells. Indeed, *R. gnavus* mono-associated mice showed induction of several genes involved in tryptophan metabolism as compared to germ-free mice^76^. As *R. gnavus* is a common species of the gut microbiota found in around 90% of adults^77^ and infants^78^, the ability of this species to produce tryptamine may influence human health.

## Tryptophan catabolites in the systemic circulation

Tryptophan catabolites may also systemically affect host physiology as they are absorbed through the intestinal epithelium and enter the bloodstream^19^ before they are excreted in the urine^10,32^. Association analyses between the human gut microbiome and ex vivo cytokine responses in whole blood upon microbial stimulations pinpointed a negative association between interferon gamma (IFNγ) production and bacterial genes responsible for the conversion of tryptophan into IE^79^, suggesting that IE has anti-inflammatory properties. Previous studies have suggested IPA as a scavenger of hydroxyl radicals^80^, and a protector against oxidative damage in different tissues^81–83^. However a recent study found that IA, but not IPA, had anti-inflammatory and anti-oxidative effects in LPS-activated human peripheral blood mononuclear cells (PBMCs) expressed as reduced IL-6 and IL-1β secretion and activation of the NRF2-ARE pathway^22^, a pathway suggested to be a therapeutic target towards prevention of neurodegenerative disorders^84^ and IBD^85^. Similarly, IAA and tryptamine attenuated pro-inflammatory cytokine responses in murine macrophage cultures and hepatocyte cultures in an AHR-dependent way^86^, suggesting that microbial tryptophan catabolites could influence inflammatory responses in the liver as well. The effects of tryptophan catabolites on production of cytokines may depend on AHR activation, since it has been shown that AHR signaling modifies Toll-like receptor (TLR)-regulated responses in human dendritic cells^87^. Adding to the complexity, it has been reported that AHR-mediated gene induction may be enhanced by SCFA^88^.

Although indoles in general appear to be beneficial, an exception to this is indoxyl sulfate (IS), which is a host-microbial co-metabolite generated from indole in the liver by the actions of cytochrome P450 enzymes, including CYP2E1, and sulfotransferase (SULT)^89^. This metabolite is typically characterized as a uremic toxin that accumulates in patients with chronic kidney disease (CKD) with serum concentrations around 100 µM compared to 2 µM in healthy adults^90,91^. IS is a potent agonist for AHR^92^, which induces tubulointerstitial fibrosis^93^, glomerular sclerosis^94^, vascular endothelial cell dysfunction^95^ and oxidative stress in endothelial cells^96^. IS is undetectable in germ-free mice, as the production of IS depends on commensal bacteria^19^. Thus, manipulation of the gut microbial tryptophan catabolism may be one strategy to lower circulating levels of IS, as recently demonstrated in gnotobiotic and conventional mice^18^. Whether diversion of intestinal tryptophan catabolism away from IS will be beneficial in renal diseases needs to be determined in future studies.

## Tryptophan catabolites in health and diseases

In light of the effects of tryptophan catabolites on various physiological processes, we discuss three areas in which tryptophan catabolites may play a vital role.

## Early life

In light of the great changes of the composition of the gut microbiota occurring during the first years of life^97,98^, which to a large extent is driven by changes in diet^99,100^, it is highly likely that the gut microbial tryptophan metabolism also changes in this period. Indeed, indole-producing *E. coli* are known to be abundant in the first week of life^101^, whereafter *Bifidobacterium* species, which are reported to be able to produce ILA^26^, start dominating the gut microbiome of breastfed infants^100–102^. Later, as complementary diet is introduced, bacterial genera including tryptophan catabolizing species such as *Lactobacillus*, *Ruminococcus, Bacteroides, Peptostreptococcus* and *Clostridium* become abundant in the gut of infants^99^. Combined with the fact that tryptophan catabolites via AHR activation modulate and educate the immune system as reviewed above, this makes microbial tryptophan catabolites in early life a very relevant area of research, which has not received much attention. In mice, AHR activation is required for the postnatal expansion of intestinal lymphoid cells expressing the transcription factor RORγt and for the formation of intestinal lymphoid follicles, which are immune system components involved in maintenance of intestinal homeostasis and resistance to infections^103^. Down these lines, it was recently shown that maternally transmitted microbially derived AHR ligands shape the early immune system of mouse offspring by increasing intestinal group 3 innate lymphoid cells and F4/80(+)CD11c(+) mononuclear cells^104^. Moreover, delivery mode and gestational age of infants are reported to affect urinary levels of metabolites belonging to the tryptophan pathway^105^. A recent study revealed that dietary protein depletion compromised adaptive immune responses and altered tryptophan amino acid homeostasis in human infant microbiota-transplanted pigs infected with human rotavirus^106^, and another study in neonatal pigs showed that formula feeding compared to sow feeding reduced enterochomaffin cell number and shifted tryptophan metabolism from serotonin to tryptamine^107^. Collectively, these studies suggest that factors such as maternal microbiota and diet, delivery mode, gestational age, infant microbiota and diet influence tryptophan metabolism in early life, which may be essential for the development of intestinal barrier functions and immune system.

## Inflammatory bowel disease

During the last decade, IBD has been one of the most studied human conditions linked to the gut microbiota. Interestingly, serum levels of tryptophan are significantly lower in IBD patients than in healthy controls, and is particularly reduced in patients with Crohn’s disease (CD) as compared to patients suffering from ulcerative colitis (UC)^108^. In addition, plasma tryptophan levels are reported to be reduced in CD^109,110^, whereas fecal tryptophan levels are elevated compared to healthy individuals^111^. These observations suggest that changes in tryptophan metabolism are involved in the etiology of IBD. In line with this, a tryptophan free diet increased susceptibility to DSS-induced inflammation in mice^112^. Importantly, not only tryptophan appears to play a role in the etiology of IBD. Also depletion of intestinal tryptophan catabolites may affect the severity of IBD, as it was recently shown that IBD patients have reduced fecal concentrations of the AHR agonist IAA^29^. Consistent with this, AHR is downregulated in the intestinal tissue of patients with IBD^113^ and AHR activation protects humanized mice against colitis by induction of regulatory T cells^114^. Furthermore, IPA is selectively diminished in circulating serum from human subjects with active colitis compared to healthy individuals^31^, and oral administration of indole, as well as IPA is found to ameliorate colonic inflammation in mice^31,115^. Gut microbial tryptophan metabolism thus holds promise as a therapeutic target in patients with IBD, provided that future research will untangle the relations between the individual tryptophan catabolites and the propensity of inflammation.

## Neurological diseases

Multiple sclerosis (MS) is a neurodegenerative autoimmune disorder. Astrocytes, a population of cells in the central nervous system (CNS), are thought to play an important role in the pathogenesis of MS. A recent study revealed that microbial catabolites of dietary tryptophan combined with type I interferon signaling activates AHR signaling in astrocytes and suppress CNS inflammation in an experimental autoimmune encephalomyelitis (EAE) animal model of MS^116^. In addition, they found decreased circulating levels of AHR agonists in individuals with MS, suggesting that imbalances in the uptake, production, or degradation of AHR agonists may contribute to the pathogenesis of MS and other autoimmune disorders^116^. In line herewith, Laquinimod, an oral drug that is currently being evaluated for the treatment of MS and Huntington’s disease (HD), a rare neurodegenerative disease, was recently shown to have anti-inflammatory effects in EAE mediated by AHR^117^. Interestingly, plasma levels of IPA were significantly lower in subjects with HD compared to healthy controls^30^. Thus, targeting the gut microbial tryptophan metabolism by modulating the endogenous gut microbiota or changing the diet may represent alternative strategies to prevent and treat MS and other neurological diseases.

## Future directions

**Fig. 3:**
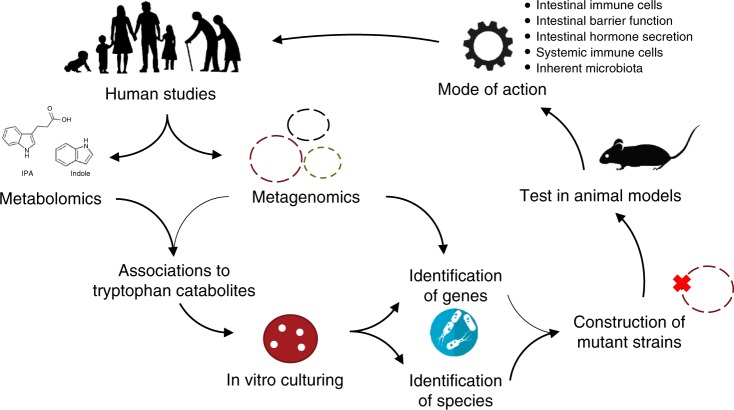
Proposed strategy to identify tryptophan-catabolite-producing microbes and to investigate their role in human health and disease. Although a number of tryptophan-catabolite producing microbes have currently been identified (Table 1), no studies have until now taken their starting point from human data, meaning that we do not know the main bacterial producers of tryptophan catabolites in the human gut. Therefore, we suggest to start from human studies and combine metagenomics and metabolomics data in order to pinpoint the most relevant and potential tryptophan-catabolite producing microbes. Based on these associations, selected target species should be cultured in the laboratory and their in vitro production of tryptophan catabolites should be assessed by growth experiments and metabolic profiling. Combined with knowledge about their genomes, this will allow the identification of genes responsible for generation of tryptophan catabolites. Knocking out identified genes of interest will subsequently allow for confirmation of the function of the gene and for testing the importance of the particular gene in relevant animal models as recently exemplified^21^. Colonizing mice with a given mutant strain and the wild-type counterpart will furthermore allow the investigation of modes of action of the tryptophan catabolites on e.g., intestinal immune cells, intestinal barrier function, intestinal hormone secretion, the inherent gut microbiota, as well as on the immune cells in systemic circulation. Together, this will provide new insights about the role of tryptophan catabolites, and lead to a better understanding of the gut microbiota in human health and disease

Although several bacteria capable of producing tryptophan catabolites have been identified (Table 1), the main contributors in the human gut remain to a large extent unknown despite the large amount of fecal metagenomics data available. As we forge ahead to determine the contributors, we propose to combine profiling of microbes (metagenomics) with quantification of tryptophan catabolites (metabolomics) in stool samples from human cohorts. This will allow us to identify important associations between tryptophan catabolites and microbial species, which can be verified using classic in vitro cultivation and metabolic phenotyping. Since tryptophan catabolites are also produced by other microorganisms than bacteria, it is imperative that we move away from profiling only the bacterial community and start exploring the human gut microbiota across kingdoms. Once we identify the relevant microorganisms and tryptophan catabolites in the gut of infants, adults and elderly, we must identify the exact role of each tryptophan catabolite in host (patho)physiology and unravel their precise mechanisms in the different segments and cells of the intestine, as well as in the different tissues by use of animal models and by conducting human interventions (Fig. 3). In addition, there is a need to identify the receptors recognizing the metabolites. Furthermore, the AHR species-dependent ligand preferences for tryptophan catabolites seen when comparing different mammals^22,47^ underline the importance of studying tryptophan catabolite interactions in human cells and not solely in murine models. Currently, the connections between tryptophan catabolites and human health remain rather tentative, with most data being associative or originating from mouse models. Therefore, a more comprehensive understanding of the dynamics of tryptophan catabolites and their functional implications in the different stages of life from birth to old age, from health to disease is needed. This may crucially extend our understanding of intestinal host-microbial cross-talk in health and disease.
